# Angiogenic and Immunomodulatory effects of embryonic stem cell derived mesenchymal stem cells in a murine model of ischemic hindlimb

**DOI:** 10.1038/s41598-025-08283-w

**Published:** 2025-07-01

**Authors:** Do Jung Kim, Young-Nam Youn, Ji Min Kim, Sang-Hyun Lim

**Affiliations:** 1 Department of Thoracic and Cardiovascular Surgery, Ajou University Medical Center, Ajou University School of Medicine, Suwon, Republic of Korea; 2 Department of Medicine, The Graduate School of Yonsei University, Seoul, Republic of Korea; 3 Department of Thoracic and Cardiovascular Surgery, Severance Cardiovascular Hospital, Yonsei University College of Medicine, Seoul, Republic of Korea; 4 Office of Biostatistics, Medical Research Collaborating Center, Ajou Research Institute for Innovation Medicine, Ajou University Medical Center, Suwon, Republic of Korea

**Keywords:** Embryonic stem cell-derived mesenchymal stem cell, Neovascularization, Immune modulation, Peripheral arterial disease, Limb-threatening ischemia, Cardiovascular diseases, Mesenchymal stem cells

## Abstract

In critical limb-threatening ischemia (CLTI), failed revascularization and pharmacotherapy substantially increase amputation and mortality risks. Mesenchymal stem cells (MSCs) are a promising therapeutic option for CLTI. This study evaluated the therapeutic effects of embryonic stem cell-derived MSCs (E-MSCs) on inflammation and angiogenesis under ischemic conditions across different E-MSC doses. Hindlimb ischemia was induced in 85 *BALB/c* nude mice by cauterizing the femoral and branched arteries. The mice were divided into five groups: non-ischemia (G1); saline-treated ischemia (G2); and ischemia treated with E-MSCs at low, medium, and high doses (G3–G5). Therapeutic effects were assessed using the rotarod test, blood perfusion ratio, and histological and cytokine analyses. G1 exhibited normal blood perfusion and motor function, whereas E-MSC-treated groups (G3–G5) demonstrated improved perfusion compared to G2. Although the medium-dose group (G4) showed numerically greater recovery, differences between G3, G4, and G5 were not statistically significant, suggesting no dose-response. All E-MSC-treated groups exhibited reduced inflammation and increases in motor function and angiogenic factors. Histological analysis revealed enhanced myofiber regeneration, reduced inflammatory infiltration, and diminished collagen deposition in the ischemic muscle of G3–G5. These changes were observed across all dose groups without significant dose-dependent differences. These results suggest E-MSCs enhance blood perfusion and modulate inflammation and angiogenesis in ischemic limbs, regardless of dose. These findings support the therapeutic potential of E-MSCs in CLTI, although further investigation is needed to optimize dosing and elucidate the mechanisms involved.

**Supplementary Information:**

The online version contains supplementary material available at 10.1038/s41598-025-08283-w.

## Introduction

Critical limb-threatening ischemia (CLTI) is a severe vascular disorder characterized by extremely insufficient blood flow, which causes tissue damage and impairs limb function^1,2^. Most patients with CLTI present with comorbidities such as diabetes and vascular diseases, which make them unsuitable for surgical interventions^3,4^. Even in cases where surgical intervention is feasible, challenges such as poor conduit vessels for bypass and inadequate patency of the proximal arteries frequently result in suboptimal surgical outcomes^5^. Furthermore, endovascular interventions using angioplasty and stenting have limited success in reducing limb amputation and mortality rates owing to high rates of restenosis^6^. Despite advances in medical treatment and revascularization procedures, current treatment options for CLTI have major limitations; innovative approaches are urgently required to enhance tissue repair and promote neovascularization.

Mesenchymal stem cells (MSCs) have emerged as a promising therapeutic option in regenerative medicine because of their unique characteristics, including self-renewal ability, immunomodulatory properties, multilineage differentiation potential, and pro-angiogenic factor-secretion ability^7,8^. Hence, MSCs are potential therapeutic candidates for various degenerative and ischemic conditions, including CLTI, myocardial infarction, and stroke^9–11^. Among the diverse sources of MSCs, embryonic stem cell-derived MSCs (E-MSCs) offer distinct advantages owing to their pluripotent nature and differentiability into cells of all three germ layers^11,12^. Moreover, E-MSCs exhibit high proliferation and expansion rates, enabling rapid growth in culture and stable immunomodulation^13,14^.

We have previously demonstrated the potential of adipose-derived MSCs (AD-MSCs) to enhance angiogenesis in a rat hindlimb ischemia model^15^. However, considering the significance of cell dosage in human clinical trials, experiments at various doses are necessary. Establishing the efficacy of MSCs and determining their minimum effective dosage could provide a substantive impetus for advancing clinical trials in patients with CLTI. Therefore, this study aimed to investigate the effects of E-MSCs on angiogenesis and inflammation in a murine model of hindlimb ischemia and to evaluate the effect of different E-MSC doses.

## Methods

Animals.

The study was approved by the Institutional Animal Care and Use Committee of the Nonclinical Research Institute, CORESTEMCEMON Inc. (Approval No.: 2023 − 0523). This study was conducted and reported in accordance with the ARRIVE guidelines. All methods were performed in accordance with relevant guidelines and regulations. Eighty-five *BALB/c* nude mice (CAnN.Cg-Foxn1nu/CrljOri; 7-week-old females, 17.6 ± 0.9 g) were purchased from Orient Bio Inc. (Seongnam, Republic of Korea) and maintained under standard conditions (22 °C ± 3 °C; 55% ± 15% humidity, 12-:12-h light/dark cycle, 150–300 lx illumination).

The mice were categorized into two main groups: non-ischemic (*n* = 17) and ischemic (*n* = 68). The non-ischemic group, designated as the sham group (G1), was not subjected to unilateral limb ischemia. The ischemic group was divided into four subgroups: G2 (control) received saline, whereas G3, G4, and G5 received low, medium, and high doses of E-MSCs (2.5 × 10^6^, 5.0 × 10^6^, and 7.5 × 10^6^ cells/kg, respectively).

### Isolation and culture of E-MSCs

Human E-MSCs from Daewoong Pharmaceutical Co., Ltd. (Seoul, Republic of Korea)^16^ were cultured in StemPro MSC SFM XenoFree medium (Gibco, Waltham, MA, USA) supplemented with 1% L-glutamine (Gibco) on Corning CellBIND surface cell culture plates (Corning Inc., Corning, NY, USA) at 37 °C. We assessed cell viability using an automated cell counter (Cedex HiRes Analyzer; Roche, Basel, Switzerland) and quantified it as a percentage of live cells relative to the total cell count. We then analyzed cell surface markers using a FACSVerse flow cytometer (BD Bioscience, Franklin Lakes, NJ, USA) and the FACSuite software (BD Bioscience).

## Hindlimb ischemic model

**Fig. 1:**
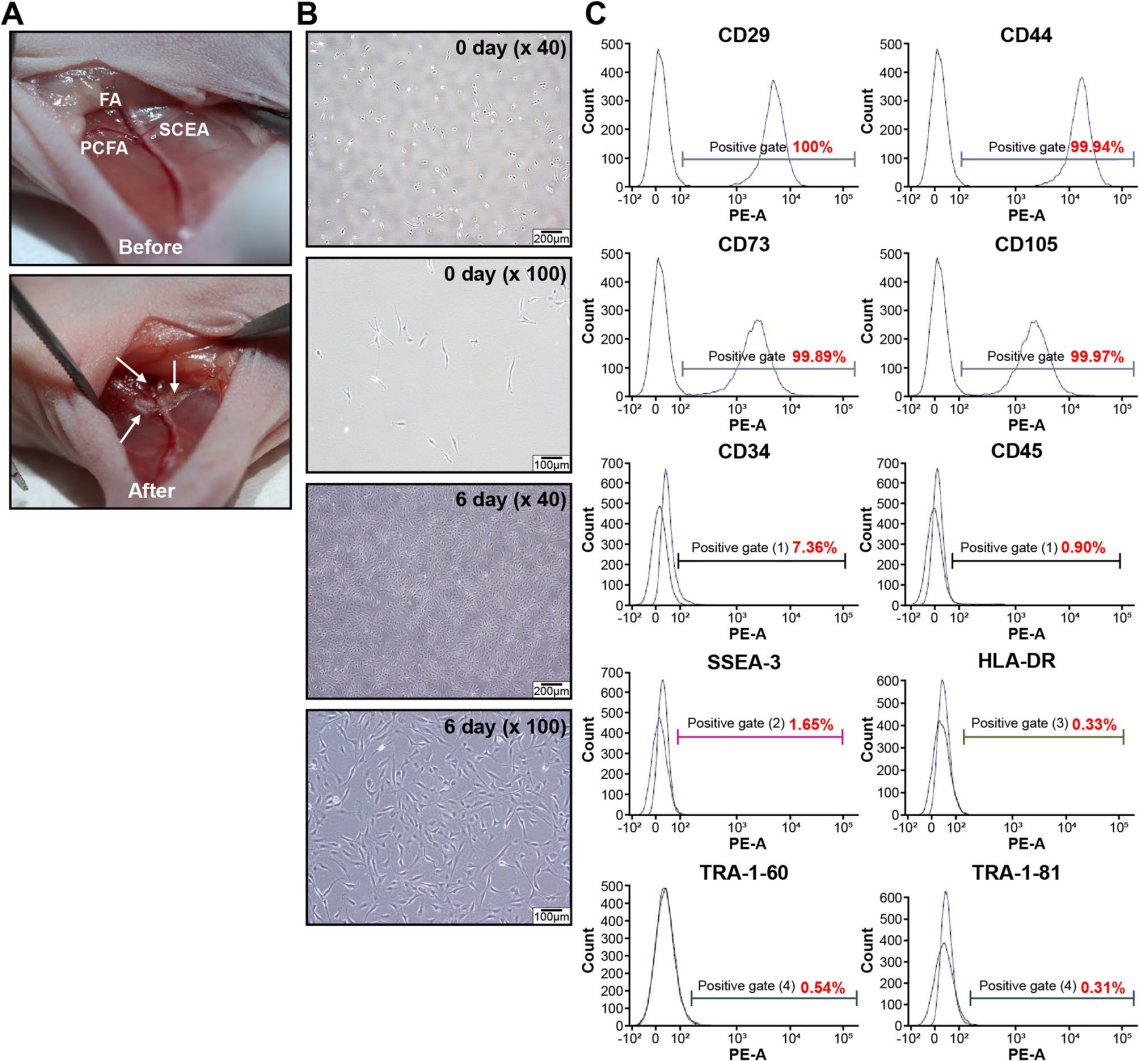
Murine ischemic model and characterization of E-MSCs. (**a**) Before and after ischemia induction (**b**) Morphology of E-MSCs after plating from days 1 to 6 (×40 and ×100 magnifications, respectively). (**c**) E-MSCs strongly expressed CD29, CD44, CD73, and CD105. The black lines indicate isotype-matched control staining. The blue lines represent cells stained with fluorescent dye-conjugated antibodies. FA, femoral artery; PCFA, proximal caudal femoral artery; SCEA, superficial caudal epigastric artery, HLA, human leukocyte antigen; PE-A, phycoerythrin-conjugated antibodies; SSEA, stage-specific embryonic antigen; TRA, tumor-related antigen.

Prior to the experiment, all mice were examined for abnormalities, and their body weights were recorded. Eighty-five mice with body weights closest to the mean were randomly selected and assigned to each group. After 8 days of acclimation, the mice were anesthetized with 3% isoflurane inhalation. The left hindlimb was disinfected with 70% ethanol. To establish a hindlimb ischemia model, a skin incision was made to expose the left iliac and femoral arteries. The femoral artery, vein, and nerve bundles were separated from the muscle tissue, and the femoral, proximal caudal femoral, and superficial caudal epigastric arteries were cauterized and excised^17^ (Fig. 1a). The incision was closed with 6–0 silk sutures. The right hindlimb served as a control. After 1–2 h, saline or E-MSCs at different doses were injected into the abductor muscle at three points around the incision.

## Necrosis assessment

Necrosis was assessed on postoperative days (PODs) 1, 3, 7, 14, and 21. After anesthesia with 3% isoflurane, necrosis severity in the ischemic hindlimb was scored on a scale of 0–4: 0 for no necrosis, 1 for minor necrosis of the nail bed, 2 for necrosis of all digits, 3 for loss of at least one digit, and 4 for severe necrosis involving two or more digits or significant foot necrosis^18^. Necrosis severity was used as an indirect measure of limb salvage, defined as the percentage of mice with severe necrosis (scores 3–4). At each time point (PODs 3, 7, 14, and 21), a subset of animals was randomly sacrificed for cytokine and histological analyses. On POD 21, necrosis scores were obtained from the remaining four mice per group.

## Rotarod test

The rotarod test was performed to evaluate motor function and balance in experimental mice before surgery and on PODs 1, 3, 7, 14, and 21. Mice were trained on a rotarod for 3 days before the first measurement. Each mouse was placed on the rotating rod, the speed of which increased from 5 to 40 rpm over 5 min. The latency time to fall was recorded, and the average of two measurements was calculated.

## Perfusion ratio measurement

Blood perfusion measurements were conducted at the same time points as the rotarod test. With the mice under anesthesia, blood perfusion in the hindlimbs was measured using a laser Doppler perfusion imaging (LDPI) system (PeriScan PIM 3; Perimed AB, Stockholm, Sweden). The LDPI system detects light scattered by blood flow to monitor the changes in hindlimb perfusion in real-time^19^. Perfusion values were measured per pixel, and images were analyzed using the LDPIwin software (version 3.1.3, Perimed AB) to calculate the blood perfusion ratio between the ischemic and non-ischemic limbs. The software was provided as part of the PeriScan PIM 3 system and was used under its standard license agreement.

### Cytokine analyses

To assess cytokine expression related to inflammation and angiogenesis, quantitative polymerase chain reaction (qPCR) was performed on PODs 3, 7, 14, and 21. Three mice per group were randomly sacrificed on PODs 3, 7, and 14. Only two mice per group were available for analysis on POD 21, as earlier time points involved interim sacrifices. The analysis focused on inflammatory cytokines [tumor necrosis factor (TNF)-α, interleukin (IL)−1β, IL-6, and transforming growth factor beta (TGF-β)] and angiogenic cytokines [angiopoietins (ANG)−1, fibroblast growth factor (FGF), vascular endothelial growth factor (VEGF), and platelet-derived growth factor (PDGF)].

Mice were euthanized under deep isoflurane anesthesia, followed by blood collection from the posterior vena cava to ensure death. Hindlimb tissues were then harvested. Total RNA was extracted using TRIzol LS Reagent (Invitrogen, Carlsbad, CA, USA). Complementary DNA (cDNA) was synthesized from the extracted RNA using the RevertAid First Strand cDNA Synthesis Kit (Thermo Fisher Scientific, Waltham, MA, USA). Real-time qPCR was conducted with the SsoAdvanced Universal SYBR Green Supermix (Bio-Rad, Hercules, CA, USA) on the CFX384 Touch Real-Time PCR Detection System (Bio-Rad). Glyceraldehyde 3-phosphate dehydrogenase (*Gapdh*) served as an internal control. Relative expression levels of target genes were calculated using the ΔΔC_T_ method^20^. Primer sequences are listed in Table 1.

**Table 1:** Sequences of primers used and quantitative polymerase chain reaction conditions.

Gene		Sequence
*IL-1Β*	forward:	5′-GAT AAC CTG GTG TGT GA-3′
	reverse:	5′-GTT CAT CTC GGA GCC TGT AG-3′
*IL-6*	forward:	5′-TCA CAA GTC GGA GGC TTA ATT ACA-3′
	reverse:	5′-TGC ACA ACT CTT TTC TCA TTT CCA-3′
*TNF-Α*	forward:	5′-CCC TGG CTG CCC TTC AC-3′
	reverse:	5′-TCA TTG AGG GCT TCT CTT GTT CT-3′
*TGF-Β1*	forward:	5′-GAG CCC TGG ATA CCA ACT AT-3′
	reverse:	5′-AGA CAG AAG TTG GCA TGG TA-3′
*ANG-1*	forward:	5′-GGG GAG GTT GGA CAG TAA TA-3′
	reverse:	5′-AGC ATG TAC TGC CTC TGA CT-3′
*FGF-2*	forward:	5′-GCT GGC TTC TAA GTG TGT TA-3′
	reverse:	5′-AGT TTA TAC TGC CCA GTT CG-3′
*VEGF*	forward:	5′-GGT TTA AAT CCT GGA GCG TT-3′
	reverse:	5′-CGT TCG TTT AAC TCA AGC TG-3′
*PDGF*	forward:	5′-CAG ATC TCT CGG AAC CTC AT-3′
	reverse:	5′-CAC ATT GCG GTT ATT GCA G-3′
*GAPDH*	forward:	5′-TGA TGG GTG TGA ACC ACG AG-3′
	reverse:	5′-GAT GGC ATG GAC TGT GGT CA-3′
RNA extraction	TRIzol LS Reagent; Invitrogen	
cDNA synthesis	RevertAid first strand cDNA synthesis kit; Thermo Fisher Scientific	
PCR reagent	SsoAdvanced Universal SYBR Green Supermix	
qPCR conditions:	95 °C, 30 s	
	95 °C, 10 s	× 40 cycles
	60 °C, 20 s	
PCR instrument	CFX384 Touch Real-Time PCR Detection System; Bio-Rad Laboratories	

qPCR, quantitative polymerase chain reaction; Il, interleukin; TNF, tumor necrosis factor; TGF, tumor growth factor; ANG, angiopoietin; FGF, fibroblast growth factor; VEGF, vascularized endothelial growth factor; PDGF, platelet-derived growth factor; GAPDH, glyceraldehyde-3-phosphate.

## Histological and immunohistochemical analyses

To evaluate muscle morphology and inflammation, sections of muscle tissues extracted on PODs 7, 14, and 21 were stained with hematoxylin and eosin (H&E). At each of these time points, two mice per group were randomly sacrificed to obtain muscle samples for histological evaluation. Hindlimb tissues were fixed in 10% buffered formalin, embedded in paraffin, and cut into 3–4-µm-thick sections. Masson’s trichrome (MT) staining was used to assess collagen deposition and fibrosis in muscle tissues.

Vascularization was evaluated using immunohistochemical (IHC) staining with anti-CD31 for capillary density and anti-α-smooth muscle actin (α-SMA) for arteriole density. Vessel density was quantified as the number of CD31^+^ or SMA^+^ cells/µm^2^ of muscle tissue. The stained sections were observed under a light microscope (Eclipse 80*i*; Nikon, Tokyo, Japan) equipped with a ProgRes C5 camera (Jenoptik Optical Systems GmbH, Jena, Germany) and a computer-assisted automated image analyzer (*i*Solution FL ver 9.1; IMT *i*-solution Inc., BC, Canada).

### Statistical analysis

Statistical analyses were performed using IBM SPSS software version 25.0 (IBM Corp., Armonk, NY, USA) and R software version 4.2.3 (R Foundation for Statistical Computing, Vienna, Austria). Data are presented as mean ± standard deviation. One-way analysis of variance (ANOVA) was performed to compare the means of continuous variables among the five groups (G1–G5). If the one-way ANOVA showed a significant difference among the groups, post-hoc tests using Bonferroni correction were conducted to identify the specific groups differing significantly from each other. Time effects were analyzed using repeated-measures ANOVA. Results with *P* < 0.05 were considered statistically significant.

## Results

### E-MSC characterization

The viability levels of E-MSCs in G3, G4, and G5 were 89.9%, 93.0%, and 96.1%, respectively (Table 2). Six days after seeding, the cells exhibited fibroblast-like morphology and adhered to the plastic culture dish, indicative of their mesenchymal nature (Fig. 1b). We confirmed the stromal characteristics of the E-MSCs through flow cytometry, which showed high expression of CD29, CD44, CD73, and CD105, along with low or no expression of hematopoietic markers (CD34 and CD45), human leukocyte antigen (HLA)-DR, and pluripotency markers (stage-specific embryonic antigen (SSEA)−3, tumor-related antigen (TRA)−1-60, and TRA-1–81) (Fig. 1c).

**Table 2:** Live and total nucleated cell counts and viability in the E-MSC-treated groups.

Variable	Group 3^a^	Group 4^a^	Group 5^a^
Number of live cells (n)	6.35 × 10^4^	1.39 × 10^5^	2.11 × 10^5^
Total cell count (n)	7.06 × 10^4^	1.5 × 10^5^	2.19 × 10^5^
Viability^b^ (%)	89.9%	93.0%	96.1%

^a^Groups 3, 4, and 5 represent cohorts administered E-MSC at low, medium, and high doses, respectively, following induction of ischemia.

^b^Cell viability (%) = (number of live cells/total cell count) × 100; E-MSC, embryonic stem cell–derived mesenchymal stem cell.

#### Necrosis severity and limb salvage rates

**Fig. 2:**
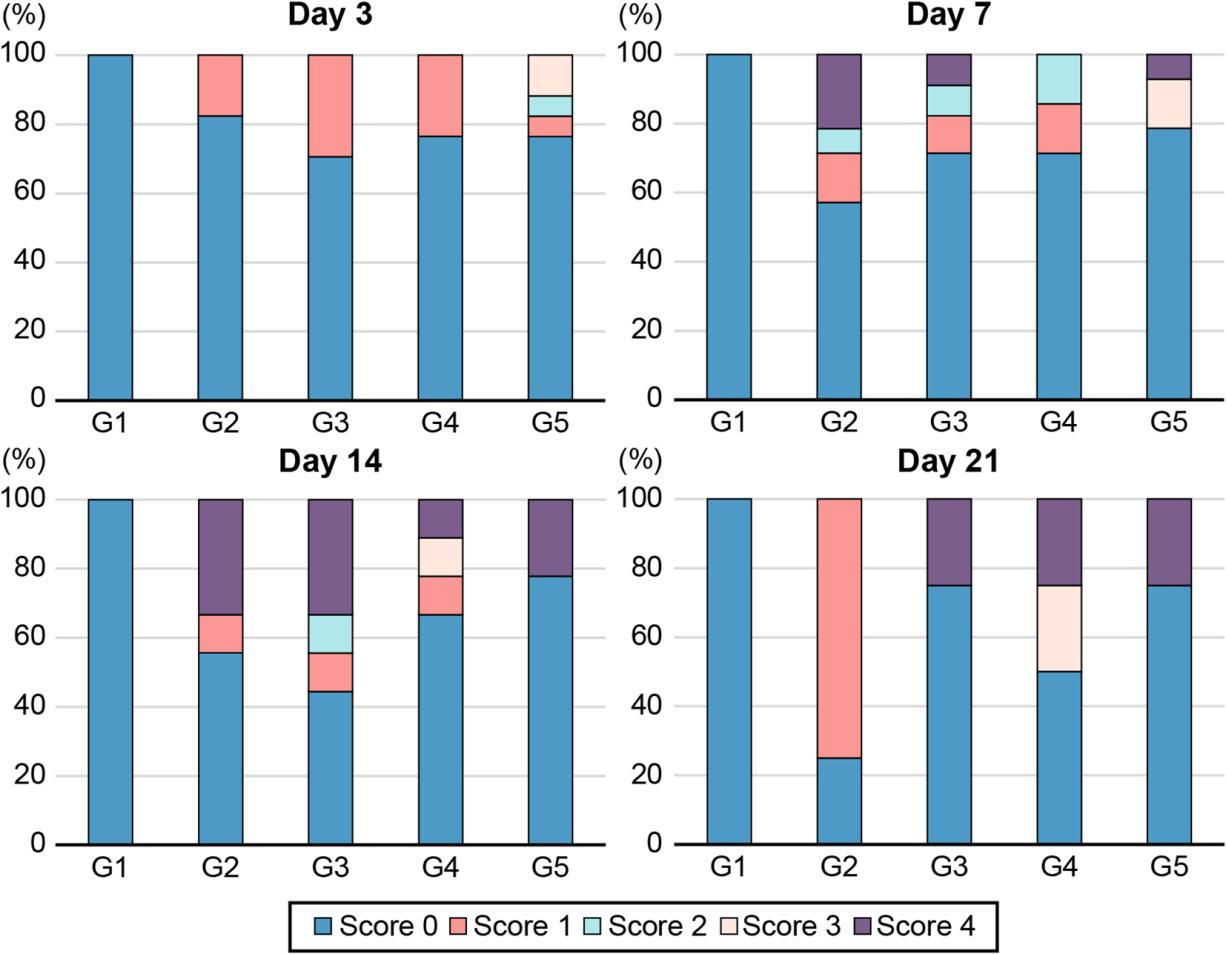
Changes in the necrosis score at each time point. Necrosis severity increased slightly over time; however, no significant differences were noted between the control and E-MSC-treated groups, nor based on E-MSC dosage. On days 3, 7, 14, and 21, the P values were 0.05, 0.029, 0.306, and 0.068, respectively.

The non-ischemic group (G1) did not develop necrosis throughout the study period. In the ischemia-induced groups (G2–G5), 28.5%, 14.3%, 14.3%, and 21.4% of mice had necrosis scores of ≥ 2 on POD 7, respectively. The corresponding limb salvage rates (defined as the percentage of mice with necrosis scores < 3) were 78.6% (G2), 85.7% (G3), 85.7% (G4), and 92.9% (G5). By POD 21, the limb salvage rates were 100.0%, 75.0%, 75.0%, and 75.0%, respectively. Among the remaining mice in the ischemia-induced groups (G2–G5), no significant changes in necrosis severity were observed over time (*P* = 0.068). Additionally, necrosis severity did not significantly differ according to E-MSC dose (Fig. 2). To further assess limb preservation, we constructed a Kaplan–Meier survival curve using a necrosis score ≥ 3 as the event threshold. Although the differences were not statistically significant (*P* = 0.32), the G2 group showed a lower trend in limb salvage probability by POD 21 (Supplementary Figure S1).

#### E-MSCs increase motor function and perfusion recovery

**Fig. 3:**
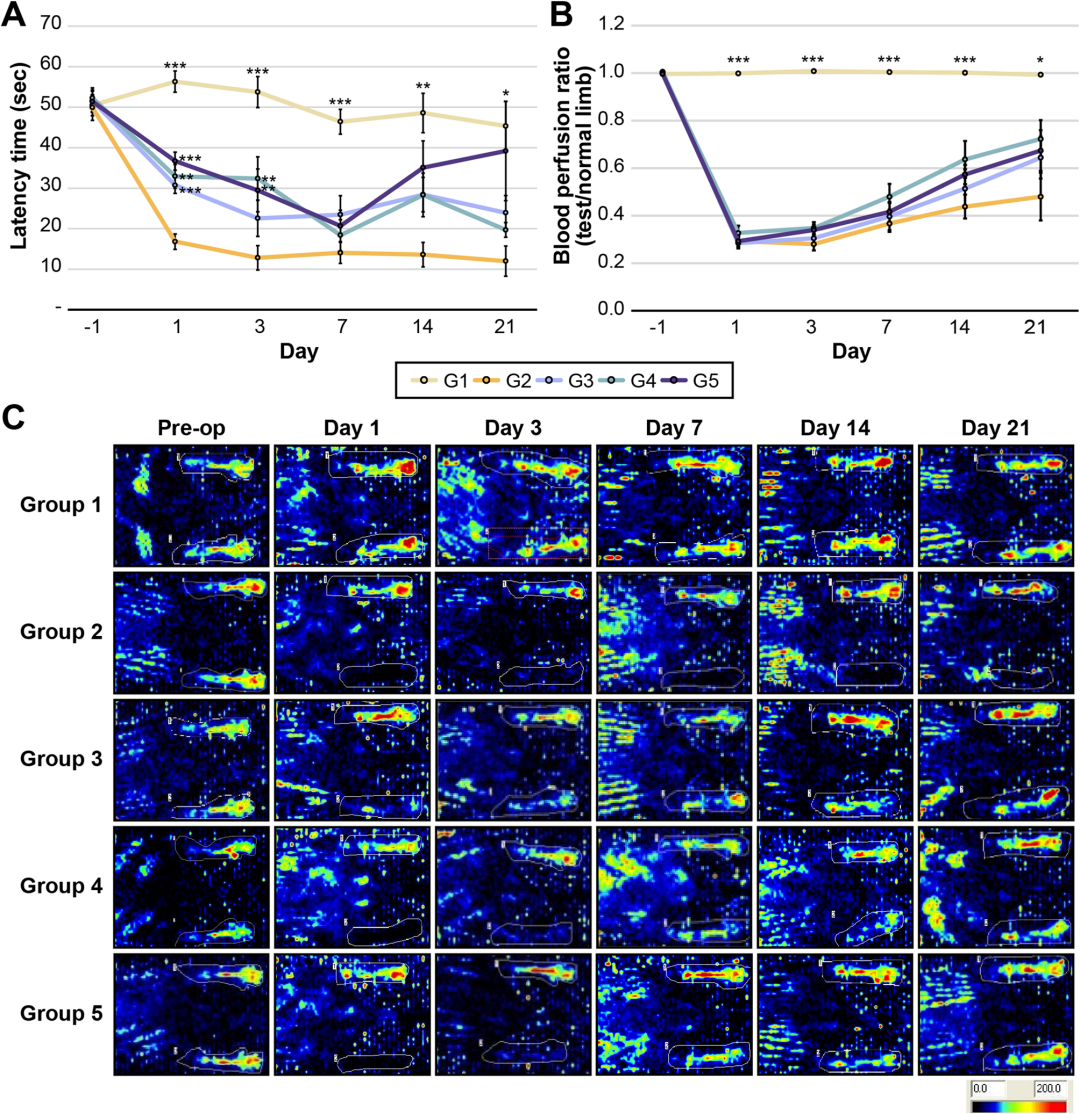
Changes in latency time and blood perfusion ratio. (**a**) After ischemia induction, the latency time in the E-MSC-treated groups (G3–G5) decreased to less than that in the control group (G2). (**b**) Compared to G1, the ischemia-induced groups (G2–G5) exhibited significantly low perfusion ratios. While G3–G5 showed a slight trend toward improved perfusion ratios compared to G2, no differences were observed based on E-MSC dosage. However, the perfusion ratios within each group (G2–G5) significantly increased over time, particularly on PODs 14–21 compared with those on POD 1 or 3 (*P* < 0.05). (**c**) Laser doppler perfusion imaging at different time points. Dark blue indicates low to no blood perfusion, whereas red represents the highest perfusion level. (*, *P* < 0.05, **, *P* < 0.01, and ***, *P* < 0.001 by the Kruskal–Wallis test).

As shown in Fig. 3a, the non-ischemic group (G1) maintained normal motor coordination and balance, with no significant changes in latency time (*P* = 0.235). The control group (G2) showed a sharp decrease in latency time on POD 1, with no signs of recovery over time. In the E-MSC-treated groups (G3–G5), the latency time decreased post-surgery but to a lesser extent compared with that in G2 (*P* < 0.05). The latency time in G3–G5 gradually improved after 7 days, although the difference was not significant among the groups.

G1 maintained a blood perfusion level comparable to that of the normal limb. In contrast, the ischemia-induced groups (G2–G5) had significantly lower perfusion ratios than G1 (*P* < 0.05). The saline-treated group (G2) showed only a minimal increase in perfusion ratio over 21 days, without significant recovery. In contrast, the E-MSC-treated groups (G3–G5) showed significant improvements in blood perfusion (*P* < 0.05). Although G4 showed the highest mean perfusion recovery numerically, no significant differences were observed among G3, G4, and G5, suggesting a lack of dose-dependent efficacy (Fig. 3b and c).

#### E-MSCs increase the secretion of angiogenic and anti-inflammatory cytokines

**Fig. 4:**
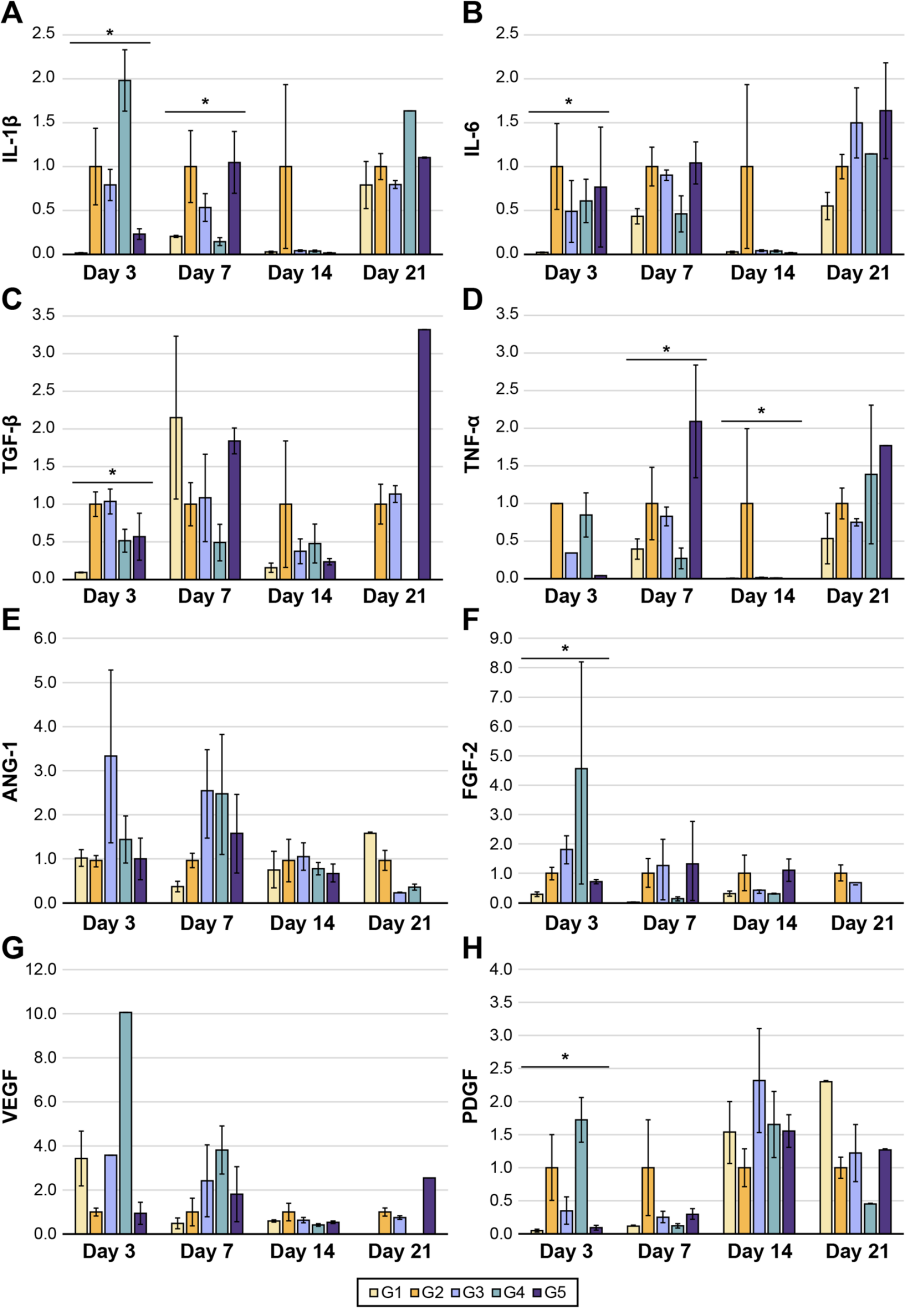
mRNA expression levels of cytokines determined using qRT-PCR at different time points. (**a–d**) Relative expression levels of anti-inflammatory cytokines (IL-1β, IL-6, TGF-β, and TNF-α). (**e–h**) Relative expression levels of angiogenic cytokines (ANG-1, FGF-2, VEGF, and PDGF) ** indicate a significant difference when compared to all groups (G1–G5), with *P* < 0.05 at the noted time points.

The control group (G2) tended to show increased levels of IL-1β, IL-6, TNF-α, and TGF-β (except for the TGF-β level on POD 7) compared to the sham group (G1) (Fig. 4a–d). The E-MSC-treated groups (G3–G5) exhibited decreased levels of these inflammatory cytokines compared to G2, although the differences were not significant. On POD 21, the expression of inflammatory cytokines increased in all groups. These results suggest that E-MSC transplantation enhances the anti-inflammation process of the injured hindlimb.

The levels of angiogenic cytokines induced by ischemia tended to be higher than that in G1, except for the level of VEGF on POD 3. The E-MSC-treated groups (G3–G5) exhibited higher early-stage mRNA expression of ANG1, FGF, VEGF, and PDGF than those of G2, indicating the role of E-MSCs in angiogenesis following ischemic injury. Over time, their expression gradually decreased in the E-MSC-treated groups (G3–G5). All groups had high levels of PDGF on PODs 14 and 21, with no significant differences among the groups (Fig. 4e–h).

#### E-MSC transplantation can mitigate ischemia-related pathologies

**Fig. 5:**
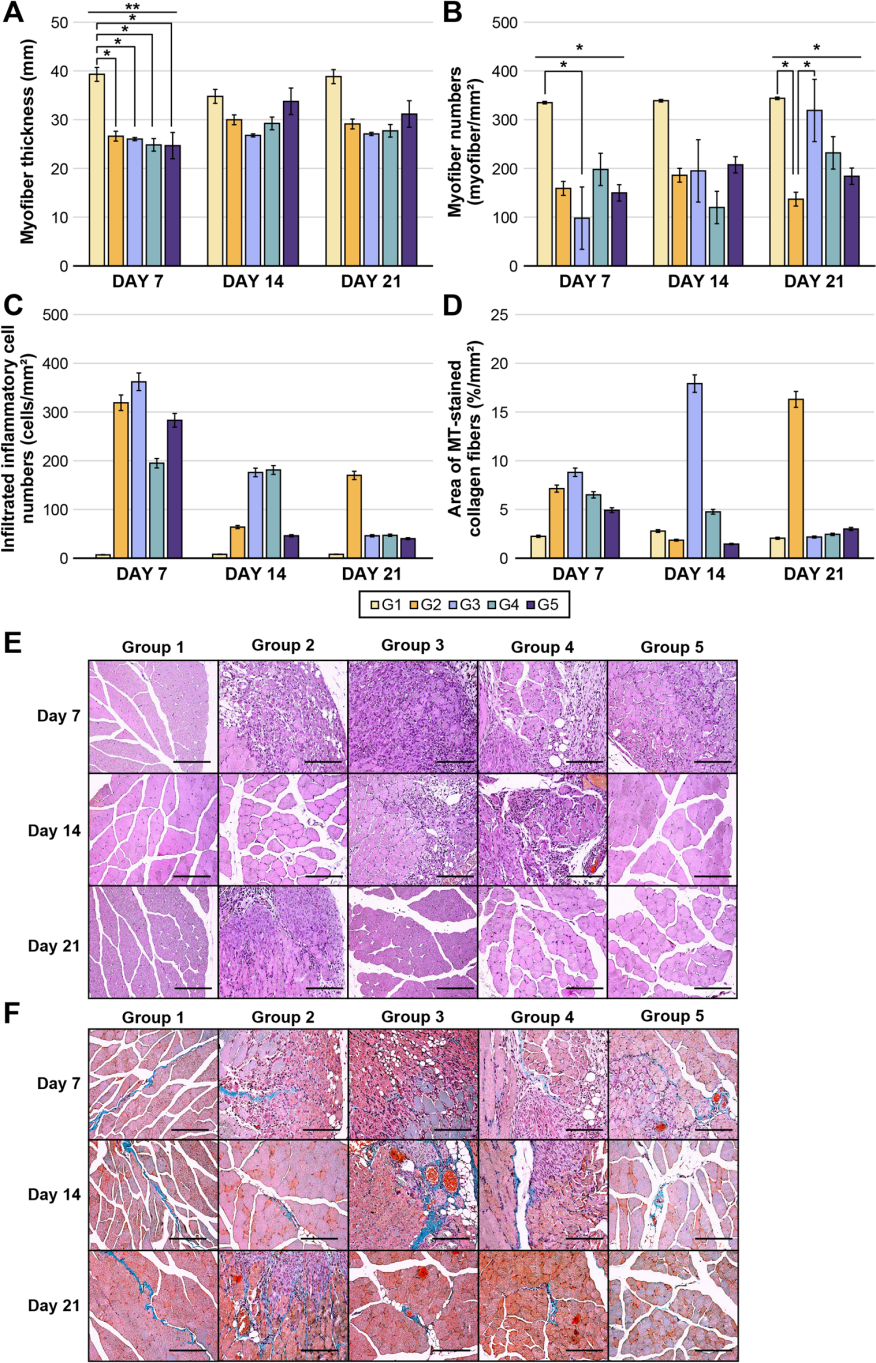
Histological analysis and staining images of muscle tissues. (**a–d**) Compared to G1, ischemia led to decreased myofiber thickness, increased muscle degeneration, and a tendency to elevate inflammation and fibrosis. On day 21, the E-MSC-treated groups showed significant muscle regeneration and a trend of reduced inflammation and fibrosis compared to G2. However, no differences were seen based on E-MSC doses. The number of myofibers/mm² was measured to illustrate the extent of muscle regeneration. (*, *P* < 0.05 and **, *P* < 0.01) (**e**) Hematoxylin and eosin (H&E) and (**f**) Masson’s trichrome (MT) staining for ischemic muscle (×200 magnification). In H&E staining, infiltrated inflammatory cells appear dark purple, whereas collagen fibers appear blue after MT staining. (Scale bar: 100 μm).

On POD 7, H&E staining showed that the ischemia-induced groups (G2–G5) had significantly reduced muscle thickness (*P* = 0.004) and substantial muscle degeneration (*P* = 0.027) compared to the G1 group, although no significant difference was observed between the G2 and E-MSC-treated groups (G3–G5) (Fig. 5a and b). Over time, the E-MSC-treated groups showed no significant difference in myofiber thickness compared to G2 but exhibited considerably greater muscle regeneration (*P* = 0.011), with no E-MSC dose-dependent differences.

The G2–G5 groups showed a trend of increased inflammatory cell proportions and fibrosis because of ischemia (Fig. 5c and d). On day 21, the E-MSC-treated groups showed a significant trend of reduced inflammation and fibrosis compared to G2, although no significant differences were observed among groups treated with E-MSC at different doses (Fig. 5e and f).

#### E-MSC treatment ameliorates vascular regeneration in the ischemic hindlimb

**Fig. 6:**
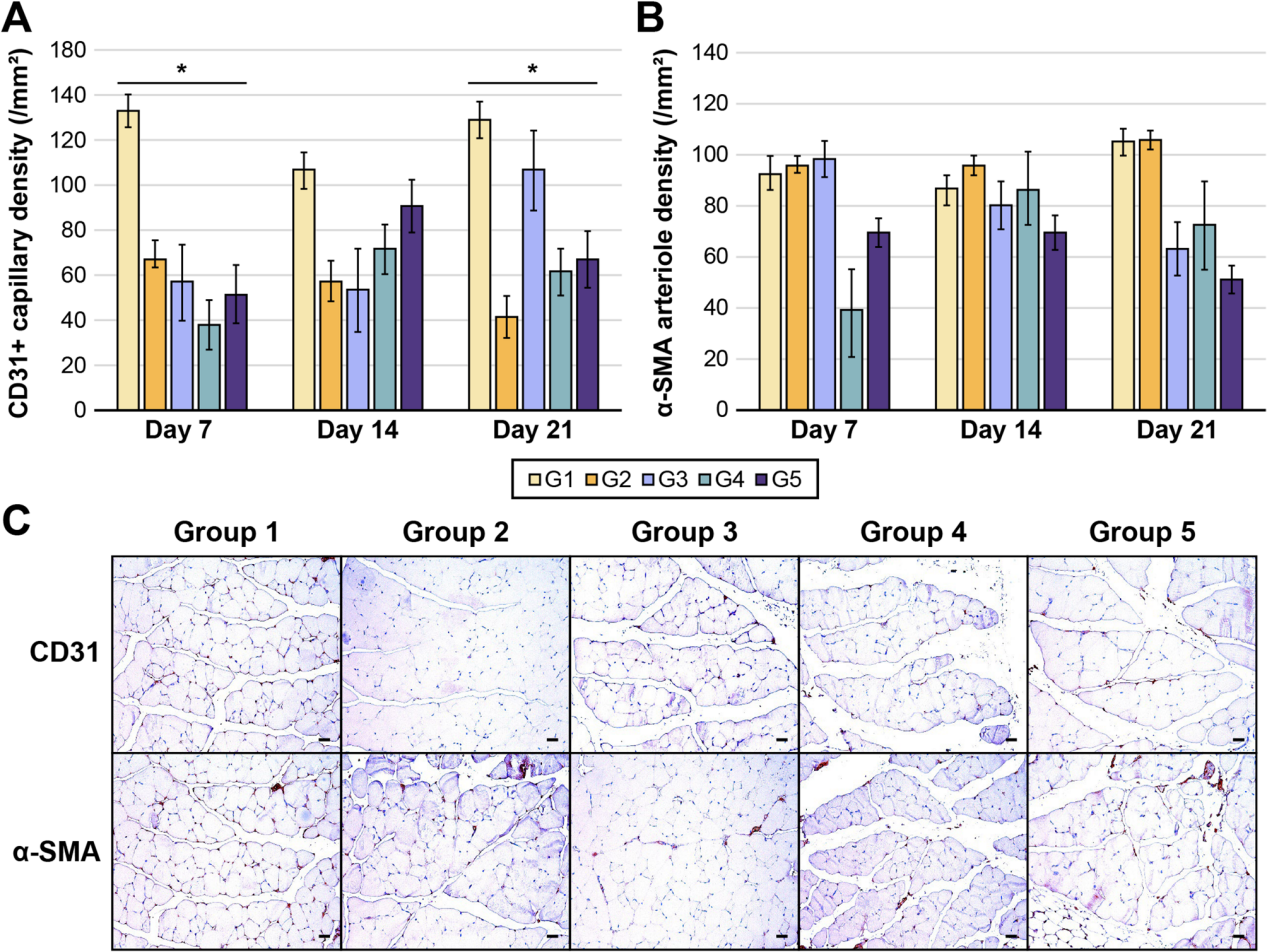
Capillary and arteriole densities in muscle tissue. (**a**) The ischemia-induced groups (G2–G5) showed a decrease in CD31^+^ capillary density compared to G1; however, no differences were noted based on the E-MSC dosage. (**b**) The α-smooth muscle actin (α-SMA) arteriole density was similar in all groups. (*, *P* < 0.05) (c) CD31 and α-SMA staining images on day 21. The capillary and arteriole appear brown in the image (×200 magnification). (Scale bar: 100 μm).

G2 exhibited decreased density of CD31^+^ capillaries compared to G1, whereas no significant changes were observed in the α-SMA^+^ arteriole density between the groups (Fig. 6a and b). However, on POD 21, compared to G2, the E-MSC-treated groups (G3–G5) showed increased CD31^+^ capillary density, suggesting that E-MSCs can lead to endothelial differentiation in the ischemic hindlimbs. Over time, the α-SMA level in the E-MSC-treated groups slightly decreased compared with that in G2, although no significant differences were observed at any time point. α-SMA expression was also observed between muscle fibers, indirectly suggesting that E-MSCs may contribute to arteriole formation in pre-existing vessels (Fig. 6c).

## Discussion

In this study, we evaluated the therapeutic effects of E-MSCs in a murine hindlimb ischemia model and investigated the potential differences based on E-MSC dose. Our findings revealed that E-MSC transplantation improved blood perfusion, enhanced motor function, and increased microvessel density in ischemic limbs. Additionally, the E-MSC-treated ischemia groups showed a substantial reduction in inflammatory cytokine (TNF-α, IL-1β, IL-6, and TGF-β) levels and a notable increase in angiogenetic cytokine (ANG1, FGF, VEGF, and PDGF) levels compared to the control group. However, discernible differences in therapeutic effects based on E-MSC dose were not observed.

Stem cell-based therapies are a promising avenue for patients who are unsuitable for surgery or have limited options for endovascular procedures^2^. With their regenerative and paracrine effects, MSCs may target the intricate pathophysiology of CLTI and other cardiovascular diseases^7,8^. Paracrine effects, mediated by the secretion of growth factors, cytokines, and extracellular vesicles, are pivotal in promoting angiogenesis, modulating inflammation, and enhancing tissue repair^21,22^. MSCs are derived from various sources, such as the umbilical cord, placenta, adipose tissue, bone marrow (BM), and induced pluripotent stem cells (iPSC)^23,24^. In this study, we focused on the angiogenic and anti-inflammatory effects of E-MSCs mediated by paracrine activity.

Several studies have shown that MSCs can alleviate tissue damage and promote functional recovery following ischemic injury^13,25,26^. In line with these results, we found that E-MSC injection recovered blood perfusion over time and improved the latency time of ischemic limbs. Restoring blood flow is crucial for tissue healing and reducing ischemia severity. In this context, the E-MSC-treated group showed better functional outcomes, with higher capillary and arteriole densities in ischemic muscle post-treatment. These findings suggest that E-MSCs can mitigate tissue damage by promoting tissue repair and neovascularization^12^. However, unlike traditional hindlimb ischemia models that often exhibit spontaneous perfusion recovery, the saline-treated group in our study demonstrated only minimal improvement. This observation could be attributed to two key factors. First, the ischemia model used in this study involved cauterization of the femoral artery and its branches, inducing more severe vascular damage than that observed in conventional ligation models. Second, the use of *BALB/c* nude mice, which lack a functional immune system, may have impaired endogenous angiogenic responses, further limiting spontaneous recovery. These factors suggest the importance of considering both ischemia severity and host immune status in preclinical evaluations of stem cell therapy.

In our study, while the group administered 5.0 × 10⁶ cells/kg E-MSCs (G4) showed the highest blood perfusion numerically, no statistically significant differences were observed among the E-MSC-treated groups (G3–G5). This suggests the lack of a clear dose–response relationship, possibly due to all tested doses exceeding a minimum effective threshold, leading to a plateau effect via paracrine mechanisms. These findings imply that lower E-MSC doses may suffice to achieve therapeutic effects, which could guide cost-effective clinical application strategies. Further studies are needed to refine optimal dosing, injection frequency, and long-term outcomes to enhance clinical translation^8,27^.

Regarding necrosis severity, the limb salvage rates did not significantly improve over time in the E-MSC-treated groups. Several factors should be considered when interpreting these findings. The reduced number of mice at later time points—due to planned interim sacrifices for molecular and histological analyses—likely limited the statistical significance of necrosis assessments, particularly on POD 21. Nevertheless, histological analyses revealed reduced inflammation and fibrosis, increased muscle regeneration, and enhanced capillary density in E-MSC-treated groups, suggesting improved tissue viability and perfusion that may not be fully captured by necrosis scoring alone. Additionally, the Kaplan–Meier curve for limb salvage demonstrated a trend toward reduced preservation in the untreated ischemic group, in line with histological observations. Collectively, these results indicate that E-MSCs may promote functional tissue preservation through paracrine mechanisms, even if overt necrosis severity does not significantly differ. Future studies with larger cohorts and complementary imaging modalities are warranted to better evaluate long-term limb salvage.

Ischemia induces tissue damage and triggers an inflammatory response as a natural defense mechanism^28^. In our study, ischemic conditions led to elevated levels of pro-inflammatory cytokines (TNF-α, IL-1β, and IL-6), which may further exacerbate tissue injury. In contrast, E-MSC-treated groups showed reduced expression of these cytokines, suggesting that E-MSCs exert anti-inflammatory effects. These immunomodulatory effects may involve multiple mechanisms, including activation of regenerative signaling cascades, suppression of pro-inflammatory transcription factors, induction of regulatory T cells, and macrophage polarization^29^. Although we did not perform mechanistic assays such as Western blotting or ELISA, previous studies provide insight into the molecular pathways likely involved. Liu et al. demonstrated that hypoxia-preconditioned MSCs activate the signal transducer and activator of transcription 3 (STAT3) and inhibit the nuclear factor kappa-light-chain-enhancer of activated B cell (NF-κB) signaling, resulting in reduced inflammatory cytokine secretion and improved tissue recovery in ischemia-reperfusion injury^30^. Similarly, Pilny et al. showed that IL-6 secreted by human AD-MSCs promotes M2 macrophage polarization, thereby enhancing muscle regeneration in ischemic environments^31^. These findings suggest that modulation of STAT3, NF-κB, and IL-6 signaling may underlie the immunoregulatory effects observed in our study. However, to translate these findings into clinical practice, long-term follow-up is essential to assess the durability, safety, and potential oncogenic risks of E-MSC therapy.

In addition, TGF-β suppresses lymphocyte activation and regulates inflammatory responses^32^. In our study, elevated TGF-β expression in the sham group on POD 7 may reflect activity-induced physiological stress, while its sustained expression in the control group likely indicates ongoing inflammation due to unresolved ischemia or delayed cytokine degradation. By POD 21, both E-MSC treated and control groups showed increased levels of inflammatory cytokines, potentially indicating a resurgence of inflammation during tissue remodeling. Given its multifaceted role in cell proliferation, differentiation, immune homeostasis, and extracellular matrix remodeling^33,34^, TGF-β may also contribute to tissue repair in E-MSC-treated limbs. These results support the potential of E-MSCs to facilitate immune regulation and tissue regeneration via multiple paracrine mechanisms.

Previous studies have indicated that MSCs promote angiogenesis primarily through the secretion of pro-angiogenic factors such as ANG-1, FGF, VEGF, and PDGF^35–37^. These factors are upregulated in response to ischemia and play key roles in recruiting endothelial cells and supporting vascular maturation^36,37^. In our study, E-MSCs enhanced the expression of VEGF, ANG-1, and FGF-2 during the early phases of ischemia, suggesting their contribution to neovascularization and tissue repair^38^. However, this effect was not sustained beyond day 21, likely due to adaptive feedback mechanisms within the ischemic microenvironment. Repeated administration of E-MSCs may therefore be necessary to maintain prolonged vascular regeneration and improve long-term perfusion.

The higher VEGF expression in the sham group compared to the control group on POD 3 may reflect transient upregulation from surgical injury and physiological stress. VEGF signaling is crucial for angiogenesis, primarily through activation of VEGF receptor 2 (VEGFR2), which initiates downstream pathways such as Akt and ERK1/2 to promote endothelial cell proliferation, migration, survival, and vascular remodeling^37,39,40^. In addition, Wang et al. demonstrated that STAT3 directly regulates VEGF expression in BM-MSCs and that STAT3 silencing significantly reduces VEGF levels^41^. Furthermore, Gangadaran et al. reported that MSC-derived extracellular vesicles directly activate VEGFR signaling, leading to improved perfusion and neovascularization in ischemic limbs^42^. These mechanistic insights support the hypothesis that the angiogenic effects observed in our study may be mediated through STAT3-dependent VEGF expression and VEGFR2-mediated vascular signaling.

Ischemia causes tissue damage and triggers cellular responses, including the upregulation of angiogenic factors and stimulation of smooth muscle and endothelial cells, aiding in tissue repair^43^. This process increases α-SMA expression, suggesting that MSCs may actively contribute to arteriole formation. Arterioles promote neovascularization and restore blood flow in ischemic tissues, which may explain the marked improvement in blood perfusion observed in the E-MSC-treated groups. However, this study indicated a slight decrease in α-SMA expression, suggesting that blood flow may have improved because of the development of existing vasculature, rather than through new vessel formation. Furthermore, positive expression of the endothelial cell marker CD31 suggests the potential differentiation of E-MSCs into endothelial-like cells in the ischemic limb. These findings demonstrate that E-MSCs may contribute to the essential process of angiogenesis, which is vital for tissue repair and regeneration.

This study had some limitations. First, immune rejection remains a potential concern, as embryonic stem cell-derived therapies may elicit host immune responses^44,45^. Although we did not observe overt signs of immune rejection or inflammation during the experimental period, the long-term immunogenicity of human-derived MSCs in allogeneic or xenogeneic settings requires further investigation. Future studies should explore strategies to enhance the engraftment and survival of transplanted E-MSCs, such as HLA modulation (e.g., HLA knockout and HLA-G overexpression), transient immunosuppression, and genetic modifications to improve immune evasion while preserving therapeutic efficacy. Second, monitoring the tumorigenic potential of embryonic stem cells is crucial^32,46^, warranting rigorous safety assessments and long-term follow-up to prevent adverse effects, including tumor formation. Third, we observed discrepancies between functional outcomes, such as motor coordination improvement and blood perfusion recovery, as well as anatomical outcomes examined through molecular analysis. Biases during the generation of the hindlimb ischemia model may have contributed to these inconsistencies. Isoflurane, known to induce ischemic preconditioning, typically safeguards tissues such as the brain, retinal, heart, and lung tissues by temporarily reducing blood flow and mitigating oxidative stress and inflammation. Thus, its effects could alter tissue responses and serve as a confounding factor, potentially distorting the observed effects of E-MSCs. Moreover, murine models may not fully reflect the complexity of human physiology. The differences in lifespan between mice (approximately 2–3 years) and humans (approximately 80 years), anatomical variations in vascular structure and hemodynamics, and variations in the degree of ischemia could also affect study outcomes. We standardized the experimental environment and conditions and ensured that tissue handling and surgical procedures were performed by a single experimenter to minimize bias.

Fourth, owing to intrinsic differences in cell expansion characteristics, culture conditions, angiogenic potential, and immunomodulatory effects, we did not directly compare E-MSCs and other MSC types in this study. However, previous literature provides indirect yet meaningful comparisons. Du et al. reported that placenta- and BM-derived MSCs exhibited superior angiogenic cytokine profiles compared to AD-MSCs in a limb ischemia model^47^. Furthermore, Lian et al. demonstrated that iPSC-derived MSCs, which share a pluripotent origin similar to E-MSCs, significantly outperformed BM-MSCs in angiogenesis and perfusion recovery^23^. Additionally, Ramkisoensing et al. reported that embryonic and fetal MSCs displayed enhanced endothelial differentiation and angiogenic potential relative to adult MSCs^48^. Collectively, these literature-based benchmarks, along with our findings of increased expression of angiogenic factors, improved perfusion, and enhanced capillary density, suggest that E-MSCs possess angiogenic capabilities comparable to or potentially exceeding those of adult MSC sources. Finally, E-MSCs in tissue were not labeled; thus, their potential to differentiate into vascular-forming cells could not be identified using molecular imaging or immunohistochemistry. Additional experiments involving repeated administrations of E-MSCs at various doses and confirmation of E-MSC viability within mice using fluorescent labeling may be warranted to clearly ascertain the therapeutic effect (paracrine effect) of E-MSCs.

## Conclusions

We established an ischemic hindlimb murine model using *BALB/c* nude mice and evaluated the potential of MSC therapy at various doses. E-MSCs are promising candidates for promoting tissue repair, angiogenesis, blood perfusion, and functional recovery under ischemic conditions, offering a new treatment option for patients with CLTI facing limited therapeutic choices. However, given that the favorable effects of E-MSCs do not endure over time, additional large-scale studies are needed to explore the therapeutic efficacy of repeated dosing and ensure the safety of E-MSCs for clinical application.

## Electronic supplementary material

Below is the link to the electronic supplementary material.

Supplementary Material 1

## Data Availability

The datasets used or analyzed during this study are available from the corresponding author on reasonable request.
