# Supplementary material for: Angiogenic and Immunomodulatory effects of embryonic stem cell derived mesenchymal stem cells in a murine model of ischemic hindlimb
Source: Sci Rep. 2025 Jul 1;15:20397. doi: 10.1038/s41598-025-08283-w (PMC12216781; doi:10.1038/s41598-025-08283-w)
Supplement: Supplementary file 1 — Supplementary Material 1 [file 41598_2025_8283_MOESM1_ESM.pdf]

Angiogenic and Immunomodulatory Effects of Embryonic Stem Cell Derived Mesenchymal Stem Cells in a Murine Model of Ischemic Hindlimb – Supplementary material

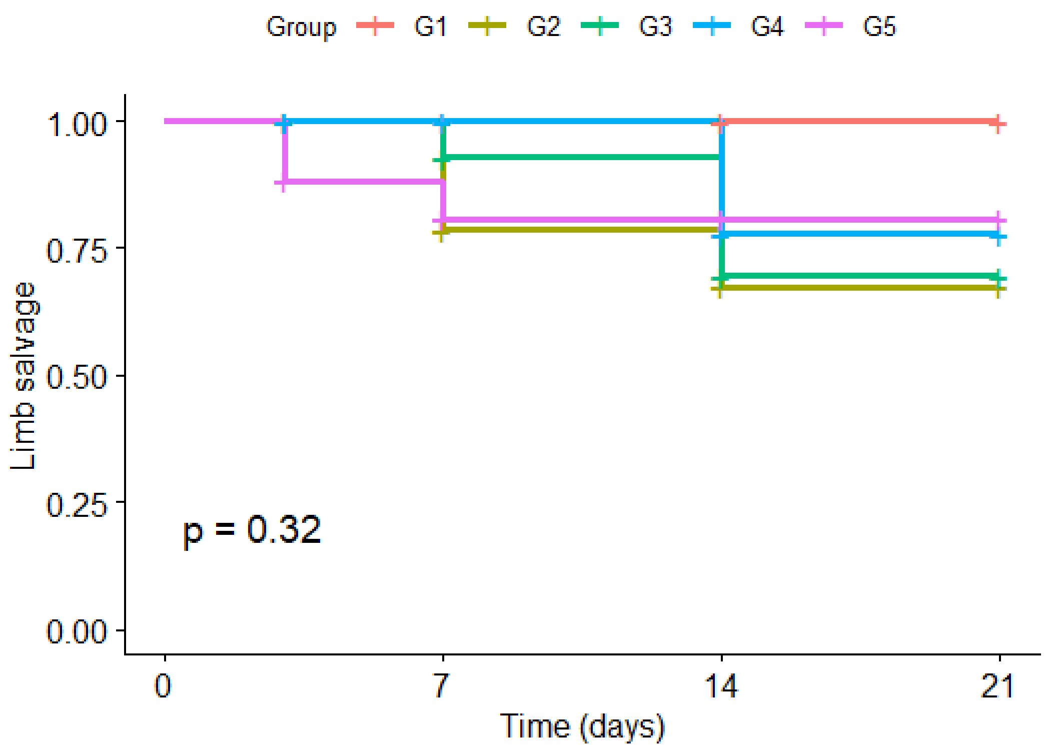

Supplementary Figure S1. Kaplan-Meier analysis of limb salvage rates in a murine hindlimb ischemia model. Limb salvage was defined as the absence of severe necrosis (necrosis score < 3) over time. The survival curve represents the proportion of mice in each group that maintained limb viability without reaching a necrosis score ≥3.
